# Applications of Artificial Intelligence to Obesity Research: Scoping Review of Methodologies

**DOI:** 10.2196/40589

**Published:** 2022-12-07

**Authors:** Ruopeng An, Jing Shen, Yunyu Xiao

**Affiliations:** 1 Brown School, Washington University in St. Louis St. Louis, MO United States; 2 Department of Physical Education, China University of Geosciences Beijing China; 3 Weill Cornell Medical College, Cornell University Ithaca, NY United States

**Keywords:** artificial intelligence, deep learning, machine learning, obesity, scoping review

## Abstract

**Background:**

Obesity is a leading cause of preventable death worldwide. Artificial intelligence (AI), characterized by machine learning (ML) and deep learning (DL), has become an indispensable tool in obesity research.

**Objective:**

This scoping review aimed to provide researchers and practitioners with an overview of the AI applications to obesity research, familiarize them with popular ML and DL models, and facilitate the adoption of AI applications.

**Methods:**

We conducted a scoping review in PubMed and Web of Science on the applications of AI to measure, predict, and treat obesity. We summarized and categorized the AI methodologies used in the hope of identifying synergies, patterns, and trends to inform future investigations. We also provided a high-level, beginner-friendly introduction to the core methodologies to facilitate the dissemination and adoption of various AI techniques.

**Results:**

We identified 46 studies that used diverse ML and DL models to assess obesity-related outcomes. The studies found AI models helpful in detecting clinically meaningful patterns of obesity or relationships between specific covariates and weight outcomes. The majority (18/22, 82%) of the studies comparing AI models with conventional statistical approaches found that the AI models achieved higher prediction accuracy on test data. Some (5/46, 11%) of the studies comparing the performances of different AI models revealed mixed results, indicating the high contingency of model performance on the data set and task it was applied to. An accelerating trend of adopting state-of-the-art DL models over standard ML models was observed to address challenging computer vision and natural language processing tasks. We concisely introduced the popular ML and DL models and summarized their specific applications in the studies included in the review.

**Conclusions:**

This study reviewed AI-related methodologies adopted in the obesity literature, particularly ML and DL models applied to tabular, image, and text data. The review also discussed emerging trends such as multimodal or multitask AI models, synthetic data generation, and human-in-the-loop that may witness increasing applications in obesity research.

## Introduction

### Background

The double burden of malnutrition, characterized by the coexistence of overnutrition (eg, overweight and obesity) and undernutrition (eg, stunting and wasting), is present at all levels of the population: country, city, community, household, and individual [1]. Obesity is a leading cause of preventable death and consumes substantial social resources in many high-income and some low- and middle-income economies [2]. Worldwide, the obesity rate has nearly tripled since 1975 [3]. In 2016, 13% of the global population, or 650 million adults, were obese [4]. More than 340 million children and adolescents aged 5 to 19 years and 39 million children aged <5 years were overweight or obese [4]. By 2025, the global obesity prevalence is projected to reach 18% among men and 21% among women [5].

Health data are now available to researchers and practitioners in ways and quantities that have never existed before, presenting unprecedented opportunities for advancing health sciences through state-of-the-art data analytics [6]. By contrast, dealing with large-scale, complex, unconventional data (eg, text, image, video, and audio) requires innovative analytic tools and computing power only available in recent years [7,8]. Artificial intelligence (AI), characterized by machine learning (ML) and deep learning (DL), has become increasingly recognized as an indispensable tool in health sciences, with relevant applications expanding from disease outbreak prediction to medical imaging and patient communication to behavioral modification [9-14]. Over the past decade, an upsurge of the scientific literature adopting AI in health research has been witnessed [15,16]. These investigations applied a wide range of AI models: from *shallow* ML algorithms (eg, decision trees (DTs) and k-means clustering) and *deep* neural networks [17] to various data sources (eg, clinical and observational) and types (eg, tabular, text, and image) [18]. This boom in AI applications raises many questions [19-21]: How do AI-based approaches differ from conventional statistical analyses? Do AI techniques provide additional benefits or advantages over traditional methods? What are the typical AI applications and algorithms applied in obesity research? Is AI a buzzword that will eventually fall out of fashion, or will the upward trend of AI adoption to study obesity continue in the future?

### Synthesizing and Disseminating AI Methodologies Adopted in Obesity Research

Three previous studies reviewed the applications of AI in weight loss interventions through diet and exercise [22-24]. They found preliminary but promising evidence regarding the effectiveness of AI-powered tools in decision support and digital health interventions [22-24]. However, to our knowledge, no study has been conducted to summarize AI algorithms, models, and methods applied to obesity research. This study remains the first methodological review on the applications of AI to measure, predict, and treat childhood and adult obesity. It serves 2 purposes: synthesizing and disseminating AI methodologies adopted in obesity research. First, we focused on summarizing and categorizing AI methodologies used in the obesity literature in the hope of identifying synergies, patterns, and trends to inform future scientific investigations. Second, we provided a high-level, beginner-friendly introduction to the core methodologies for interested readers, aiming to facilitate the dissemination and adoption of various AI techniques.

## Methods

The scoping review was conducted in accordance with the PRISMA-ScR (Preferred Reporting Items for Systematic Reviews and Meta-Analyses extension for Scoping Reviews) guidelines [25].

### Study Selection Criteria

Studies that met all of the following criteria were included in the review: (1) study design: experimental or observational studies; (2) analytic approach: use of AI, including ML and DL (ie, deep neural networks), in measuring, predicting, or intervening obesity-related outcomes; (3) study participants: humans of all ages; (4) outcomes: obesity or body weight status (eg, BMI, body fat percentage [BFP], waist circumference [WC], and waist-to-hip ratio [WHR]); (5) article type: original, empirical, and peer-reviewed journal publications; (6) time window of search: from the inception of an electronic bibliographic database to January 1, 2022; and (7) language: articles written in English.

Studies that met any of the following criteria were excluded from the review: (1) studies focusing on outcomes other than obesity (eg, diet, physical activity, energy expenditure, and diabetes); (2) studies that used a rule-based (*hard-coded*) approach rather than example-based ML or DL; (3) articles not written in English; and (4) letters, editorials, study or review protocols, case reports, and review articles.

### Search Strategy

A keyword search was performed in 2 electronic bibliographic databases: PubMed and Web of Science. The search algorithm included all possible combinations of keywords from the following two groups: (1) “artificial intelligence,” “computational intelligence,” “machine intelligence,” “computer reasoning,” “machine learning,” “deep learning,” “neural network,” “neural networks,” or “reinforcement learning” and (2) “obesity,” “obese,” “overweight,” “body mass index,” “BMI,” “adiposity,” “body fat,” “waist circumference,” “waist to hip,” or “waist‐to‐hip.” The Medical Subject Headings terms “artificial intelligence” and “obesity” were included in the PubMed search. Multimedia Appendix 1 documents the search algorithm used in PubMed. Two coauthors of this review independently conducted title and abstract screening on the articles identified from the keyword search, retrieved potentially eligible articles, and evaluated their full texts. The interrater agreement between the 2 coauthors was assessed with Cohen kappa (κ=0.80). Discrepancies were resolved through discussion.

### Data Extraction and Synthesis

A standardized data extraction form was used to collect the following methodological and outcome variables from each included study: authors; year of publication; country; data collection period; study design; sample size; training, validation, and test set size; sample characteristics; the proportion of female participants; age range; AI models used; input data source; input data format; input features; outcome data type; outcome measures; unit of analysis; main study findings; and implications for the effectiveness and usefulness of AI in measuring, predicting, or intervening obesity-related outcomes.

### Methodological Review

We classified AI methodologies adopted by the included studies into 2 primary categories: ML and DL models. Among the ML models, methods were organized into 2 subcategories: unsupervised and supervised learning. Among the DL models, methods were classified into 3 subcategories: tabular data modeling, computer vision (CV), and natural language processing (NLP). Rather than enumerating every single model performed by the included studies, which is unnecessary and unilluminating, we focused on the popular models used by multiple studies.

## Results

### Identification of Studies

**Figure 1:**
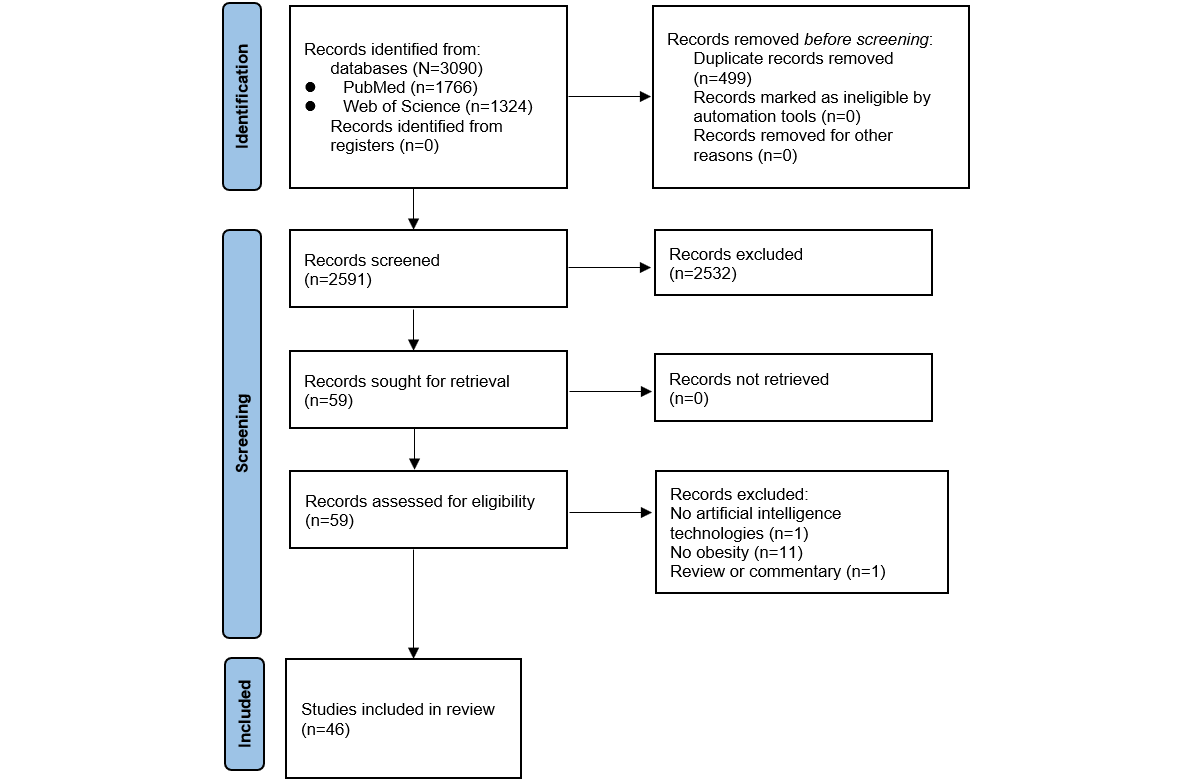
Identification of studies via databases and registers.

Figure 1 shows the PRISMA (Preferred Reporting Items for Systematic Reviews and Meta-Analyses) flow diagram. We identified a total of 3090 articles through the keyword search, and after removing 499 (16.15%) duplicates, 2591 (83.85%) unique articles underwent title and abstract screening. Of these 2591 articles, 2532 (97.72%) were excluded, and the full texts of the remaining 59 (2.28%) were reviewed against the study selection criteria. Of these 59 articles, 13 (22%) were excluded. The reasons for exclusion were as follows: no adoption of AI technologies (1/13, 8%), no obesity-related outcomes (11/13, 85%), and commentary rather than original empirical research (1/13, 8%). Therefore, of the 3090 articles identified initially through the keyword search, 46 (1.49%) were included in the review [26-71].

### Study Characteristics

Table 1 summarizes the key characteristics of the 46 included studies. An increasing trend in relevant publications was observed. The earliest study included in the review was published in 1997; others were published in, or after, 2008; for example, 2% (1/46) each in 2008, 2012, and 2017; 4% (2/46) each in 2014 and 2016; 7% (3/46) each in 2009 and 2015; 9% (4/46) in 2018; 15% (7/46) in 2019; 20% (9/46) in 2020; and 26% (12/46) in 2021. Of the 46 studies, 16 (35%) were conducted in the United States [28,32,33,37,42,46,48, 50-53,57,58,60,62,63]; 6 (13%) in China [39,40,45,56,64,65]; 3 (7%) each in the United Kingdom [27,68,69] and Korea [35,43,49]; 2 (4%) each in Italy [36,71], Turkey [41,70], Finland [44,59], Germany [54,55], and India [36,71]; and 1 (2%) each in Saudi Arabia [26], Iran [67], Serbia [66], Portugal [61], Spain [47], Singapore [38], Australia [34], and Indonesia [29]. Of the 46 studies, 32 (70%) adopted a cross-sectional study design [26,27,29-32,37,39-42,46-50,52,55-58,60-63,65-71], 7 (15%) a prospective study design [28,33,38,43,45,54,59], 6 (13%) a retrospective study design [34-36,51,53,64], and 1 (2%) a cotwin control design [44]. Sample sizes varied substantially across the included studies, ranging from 20 to 5,265,265. Of the 46 studies, 7 (15%) had a sample size of between 20 and 82; 11 (24%) between 130 and 600; 19 (41%) between 1061 and 9524; 6 (13%) between 16,553 and 49,805; 2 (4%) between 244,053 and 618,898; and 1 (2%) study had a sample size of 5,265,265. Of the 46 studies, 23 (50%) focused on adults, 14 (30%) on children and adolescents, 1 (2%) on people of all ages, and the remaining 8 (17%) did not report the age range of participants.

**Table 1:** Characteristics of the studies included in the review.

Authors, year	Country	Data collection period	Study design	Sample size	Training set size	Validation set size; test set size	Sample characteristics	Female participants (%)	Age (years)	AI^a^ model
Abdel-Aal and Mangoud [26], 1997	Saudi Arabia	1995	Cross-sectional	1100	800	N/A; 300	Patients	N/A^b^	≥20	NN^c^ (AIM^d^ abductive)
Positano et al [71], 2008	Italy	N/A	Cross-sectional	20	N/A	N/A	Participants with varying levels of obesity	N/A	Mean 52 (SD 16)	Fuzzy c-means
Ergün [70], 2009	Turkey	N/A	Cross-sectional	82	41	N/A; 41	Participants with different ranges of obesity	N/A	N/A	LR^e^, MLP^f^
Yang et al [69], 2009	United Kingdom	N/A	Cross-sectional	507	N/A	N/A	Patients	N/A	N/A	SVM^g^
Zhang et al [68], 2009	United Kingdom	1988 to 2003	Cross-sectional	16,553	11,091	N/A; 5462	Children	N/A	Birth to 3	NB^h^, SVM, DT^i^, NN
Heydari et al [67], 2012	Iran	2010	Cross-sectional	414	248	N/A; 104	Healthy military personnel	N/A	Mean 34.4 (SD 7.5)	NN, LR
Kupusinac et al [66], 2014	Serbia	N/A	Cross-sectional	2755	1929	413; 413	Adults	48.3	18 to 88	NN
Shao [65], 2014	China	N/A	Cross-sectional	248	174	N/A; 74	N/A	N/A	N/A	MR^j^, MARS^k^, SVM, NN
Chen et al [64], 2015	China	N/A	Retrospective	476	N/A	N/A	Participants with different ranges of obesity	62.4	22 to 82	NN (ELM^l^)
Dugan et al [63], 2015	United States	N/A	Cross-sectional	7519	6767	N/A; 752	Children	49	2 to 10	DT, RF^m^, NB, NN (BN^n^)
Nau et al [62], 2015	United States	2010	Cross-sectional	22,497	15,073	N/A; 7424	Children	N/A	10 to 18	RF
Almeida et al [61], 2016	Portugal	2009 to 2013	Cross-sectional	3084	1537	N/A; 664	School-age children	49.7	9	LR, NN
Lingren et al [60], 2016	United States	N/A	Cross-sectional	428	257	N/A; 86	Children	N/A	1 to 6	SVM, NB
Seyednasrollah et al et al [59], 2017	Finland	1980 to 2012	Prospective	2262	1625	N/A; 637	Adults	N/A	≥18	GB^o^
Hinojosa et al [58], 2018	United States	2003 to 2007	Cross-sectional	5,265,265	N/A	N/A	School-age children: grades 5, 7, and 9	N/A	N/A	RF
Maharana and Nsoesie [57], 2018	United States	2017	Cross-sectional	1695	508	N/A; 339	Adults	N/A	≥18	NN (CNN^p^)
Wang et al [56], 2018	China	2014 to 2015	Cross-sectional	139	111	N/A; 28	Participants with different ranges of obesity	36.7	27 to 53	SVM, KNN^q^, DT, LR
Duran et al [55], 2018	Germany	1999 to 2004	Cross-sectional	1999	1333	N/A; 666	Children	42.8	8 to 19	NN
Gerl et al [54], 2019	Germany	2012; 1991 to 1994	Prospective	1061	796	206; 250	N/A	53.8	N/A	Cubist, LASSO^r^, PLS^s^, GB, RF, LM^t^
Hammond et al [53], 2019	United States	2008 to 2016	Retrospective	3449	482	N/A; 207	Children	49.2	4.5 to 5.5	LASSO, RF, GB
Hong et al [52], 2019	United States	2008	Cross-sectional	1237	1400	N/A; 600	Patients	N/A	≥18	LR, SVM, DT, RF
Ramyaa et al [51], 2019	United States	1993 to 1994	Retrospective	48,508	33,956	N/A; 14,552	Postmenopausal women	100	50 to 79	SVM, KNN, DT, PCA^u^, RF, NN
Scheinker et al [50], 2019	United States	2018	Cross-sectional	3138	N/A	N/A	Census population	49.9	All ages	LM, GB
Shin et al [49], 2019	Korea	N/A	Cross-sectional	163	143	N/A; 20	Amateur athletes	37.4	17 to 25	NN
Stephens et al [48], 2019	United States	N/A	Cross-sectional	23	N/A	N/A	Youth with obesity symptoms	57	Range 9.78-18.54	NN
Blanes-Selva et al [47], 2020	Spain	N/A	Cross-sectional	49,805	39,844	N/A; 9961	Patients	N/A	N/A	PU^v^ learning
Dunstan et al [46], 2020	United States	2008	Cross-sectional	79	N/A	N/A	Adults	N/A	≥20	SVM, RF, GB
Fu et al [45], 2020	China	1999 to 2003	Prospective	2125	1143	381; 382	Children	40.6	4 to 7	GB
Kibble et al [44], 2020	Finland	N/A	Cotwin control	43	N/A	N/A	Young adult monozygotic twin pairs	53	22 to 36	GFA^w^
Park et al [43], 2020	Korea	N/A	Prospective	76	75	N/A; 1	Adolescents	6.8; N/A	Mean 11.94 (SD 3.13); mean 13.42 (SD 3.25)	LASSO
Phan et al [42], 2020	United States	2017 to 2018	Cross-sectional	18,700 images	14,960	N/A; 3740	Adolescents and adults	N/A	N/A	LM, NN (CNN)
Taghiyev et al [41], 2020	Turkey	2019	Cross-sectional	500	325	N/A; 175	Female patients	100	≥18	DT, LR
Xiao et al [40], 2020	China	2007 to 2010	Cross-sectional	9524	N/A	N/A	Residents	54	≥18	LR, NN (CNN)
Yao et al [39], 2020	China	N/A	Cross-sectional	67; 24	N/A	N/A	Smartphone users	N/A; 41.7	Mean 25.19; range 18-46	NN
Alkutbe et al [27], 2021	United Kingdom	2014; 2015 to 2016	Cross-sectional	1223	977	N/A; 246	Children	61.8	8 to 12	GB
Bhanu et al [38], 2021	Singapore	2003 to 2006	Prospective	130	104	N/A; 26	Older adults	69.5	Mean 67.85 (SD 7.90)	NN (U-Net)
Cheng et al [37], 2021	United States	2003 to 2004; 2005 to 2006	Cross-sectional	7162	N/A	N/A	Adults	48.6	20 to 85	NB, KNN, MEFC^x^, DT, NN (MLP)
Delnevo et al [36], 2021	Italy	N/A	Retrospective	221	176	N/A; 45	Participants with different ranges of obesity	N/A	N/A	GB, RF
Lee et al [35], 2021	Korea	2015 to 2020	Retrospective	3159	2370	N/A; 789	Obstetric patients and their newborns	100	20 to 44	LM, RF, NN
Lin et al [34], 2021	Australia	2010 to 2019	Retrospective	2495	882	N/A; 1613	Participants with different ranges of obesity	67.4	21 to 36	Two-step cluster analysis, k-means
Pang et al [33], 2021	United States	2009 to 2017	Prospective	27,203	21,762	N/A; 5441	Children	49.2	<2	DT, NB, LR, SVM, GB, NN
Park et al [32], 2021	United States	2014 to 2016	Cross-sectional	5000 tweets	4500	N/A; 500	Twitter users	60.7	Mean 51.91 (SD 17.20)	NB, SVM, NN (CNN, LSTM^y^)
Rashmi et al [31], 2021	India	2020	Cross-sectional	600 images	420	120; 60	Children	50	8 to 11	SVM, NB, RF
Snekhalatha and Sangamithirai [30], 2021	India	N/A	Cross-sectional	2700 images	2000	500; 200	Adults	N/A	Mean 45 (SD 2.5)	NN (VGG, ResNet, DenseNet)
Thamrin et al [29], 2021	Indonesia	2018	Cross-sectional	618,898	557,008	N/A; 61,890	Adults	N/A	≥18	DT, NB, LR
Zare et al [28], 2021	United States	2003 to 2019	Prospective	244,053	162,702	N/A; 81,351	Children	49	5 to 6	DT, LR, RF, NN

^a^AI: artificial intelligence.

^b^N/A: not applicable.

^c^NN: neural network.

^d^AIM: abductory induction mechanism.

^e^LR: logistic regression.

^f^MLP: multilayer perceptron.

^g^SVM: support vector machine.

^h^NB: naïve Bayes.

^i^DT: decision tree.

^j^MR: multiple regression.

^k^MARS: multivariate adaptive regression splines.

^l^ELM: extreme learning machine.

^m^RF: random forest.

^n^BN: BayesNet.

^o^GB: gradient boosting.

^p^CNN: convolutional neural network.

^q^KNN: k-nearest neighbor.

^r^LASSO: least absolute shrinkage and selection operator.

^s^PLS: partial least squares.

^t^LM: linear model.

^u^PCA: principal component analysis.

^v^PU: positive and unlabeled.

^w^GFA: group factor analysis.

^x^MEFC: multiobjective evolutionary fuzzy classifier.

^y^LSTM: long short-term memory.

### Data Sources and Outcome Measures

Table 2 summarizes the data sources and outcome measures of the studies included in the review. Input data were obtained from a variety of sources, including health surveys (eg, National Health and Nutrition Examination Survey), electronic health records, magnetic resonance imaging (MRI) scans, social media data (eg, tweets), and geographically aggregated data sets (eg, InfoUSA and Dun & Bradstreet). Of the 46 studies, 34 (74%) analyzed tabular data (eg, spreadsheet data) [26-29,33-37,39,41,44-47,49-51,53-56,58-68,70], 8 (17%) analyzed digital image data [30,31,38,40,42,43,57,71], and 4 (9%) analyzed text data [32,48,52,69]. Obesity-related measures used across the studies included anthropometrics (eg, body weight, BMI, BFP, WC, and WHR) and biomarkers.

**Table 2:** Data sources and measures of outcomes in the studies included in the review.

Authors, year	Input data source	Input data format	Input features (independent variables)	Outcome data type	Outcome measures	Unit of analysis
Abdel-Aal and Mangoud [26], 1997	Medical survey data	Tabular	13 health parameters	Continuous	WHR^a^	Individual
Positano et al [71], 2008	MRI^b^	Image	Subcutaneous adipose tissue and visceral adipose tissue	Binary	Abdominal adipose tissue distribution	Individual
Ergün [70], 2009	Obtained from participants	Tabular	24 obesity parameters	Binary	Classification of obesity	Individual
Yang et al [69], 2009	Clinical data	Text	Clinical discharge summaries	Binary	Obesity status	Individual
Zhang et al [68], 2009	Objective measure	Tabular	Data recorded regarding the weight of the child during the first 2 years of the child’s life	Binary	Obesity	Individual
Heydari et al [67], 2012	Questionnaire and objective measure	Tabular	Age, systole, diastole, weight, height, BMI, WC^c^, HC^d^, and triceps skinfold and abdominal thicknesses	Binary	Obesity	Individual
Kupusinac et al [66], 2014	Objective measure	Tabular	Gender, age, and BMI	Continuous	BFP^e^	Individual
Shao [65], 2014	Objective measure	Tabular	13 body circumference measurements	Continuous	BFP	Individual
Chen et al [64], 2015	Objective measure	Tabular	18 blood indexes and 16 biochemical indexes	Continuous	Overweight	Individual
Dugan et al [63], 2015	Questionnaire and objective measure	Tabular	167 clinical data attributes	Continuous	Obesity	Individual
Nau et al [62], 2015	Two secondary data sources (InfoUSA and Dun & Bradstreet)	Tabular	44 community characteristics	Binary	Obesogenic and obesoprotective environments	Community
Almeida et al [61], 2016	Objective measure	Tabular	Age, sex, BMI *z* score, and calf circumference	Continuous	BFP	Individual
Lingren et al [60], 2016	EHR^f^	Tabular	EHR data	Binary	Obesity	Individual
Seyednasrollah et al [59], 2017	Objective measure	Tabular	Clinical factors and genetic risk factors	Binary	Obesity	Individual
Hinojosa et al [58], 2018	Objective measure	Tabular	School environment	Binary	Obesity	School
Maharana and Nsoesie [57], 2018	Objective measure	Image	Built environment	Continuous	Prevalence of obesity	Census tract
Wang et al [56], 2018	Objective measure	Tabular	Single-nucleotide polymorphisms	Binary	Obesity risk	Individual
Duran et al [55], 2018	NHANES^g^	Tabular	Age, height, weight, and WC	Binary	Excess body fat	Individual
Gerl et al [54], 2019	Objective measure	Tabular	Human plasma lipidomes	Binary and continuous	Obesity: BMI, WC, WHR, and BFP	Individual
Hammond et al [53], 2019	EHR and publicly available data	Tabular	EHR data	Binary and continuous	Obesity status	Individual
Hong et al [52], 2019	EHR	Text	Discharge summaries	Binary	Identification of obesity	Individual
Ramyaa et al [51], 2019	Questionnaire	Tabular	Energy balance components	Binary and continuous	Energy stores: body weight	Individual
Scheinker et al [50], 2019	2018 Robert Wood Johnson Foundation County Health Rankings	Tabular	Demographic factors, socioeconomic factors, health care factors, and environmental factors	Continuous	Obesity prevalence	County
Shin et al [49], 2019	Objective measure	Tabular	Upper body impedance and lower body anthropometric data	Continuous	BFP	Individual
Stephens et al [48], 2019	From recorded dialogue	Text	Dialogue	Binary	Weight management program	Individual
Blanes-Selva et al [47], 2020	EHR of HULAFE^h^	Tabular	32 variables	Binary	Identification of obesity	Individual
Dunstan et al [46], 2020	Euromonitor data set	Tabular	National sales of a small subset of food and beverage categories	Continuous	Nationwide obesity prevalence	Country
Fu et al [45], 2020	Clinical data	Tabular	Demographic characteristics, maternal anthropometrics, perinatal clinical history, laboratory tests, and postnatal feeding practices	Binary	Obesity	Individual
Kibble et al [44], 2020	Clinical data	Tabular	42 clinical variables	Binary	Mechanisms of obesity	Individual
Park et al [43], 2020	Openly accessible database	Image	Neuroimaging biomarkers	Continuous	BMI	Individual
Phan et al [42], 2020	Objective measure	Image	Neighborhood built environment characteristics	Binary, continuous	Obesity	State
Taghiyev et al [41], 2020	EHR	Tabular	Results of blood tests	Binary	Obesity	Individual
Xiao et al [40], 2020	Objective measure	Image	Vertical greenness level	Binary	Obesity	Individual
Yao et al [39], 2020	Objective measure	Tabular	Characteristics of body movement captured by smartphone’s built-in motion sensors	Continuous	BMI	Individual
Alkutbe et al [27], 2021	Self-reported and objective measures	Tabular	Weight, height, age, and gender	Binary and continuous	BFP	Individual
Bhanu et al [38], 2021	MRI	Image	SAT^i^ and VAT^j^	Binary	Abdominal fat	Individual
Cheng et al [37], 2021	Objective measure	Tabular	Physical activity	Binary	Obesity	Individual
Delnevo et al [36], 2021	Questionnaire	Tabular	Positive and negative psychological variables	Binary and continuous	BMI values and BMI status	Individual
Lee et al [35], 2021	Objective measure	Tabular	64 independent variables: nationwide multicenter ultrasound data and maternal and delivery information	Continuous	BMI	Individual
Lin et al [34], 2021	Objective measure	Tabular	Key clinical variables	Binary	Obesity classification criterion	Individual
Pang et al [33], 2021	EHR data from pediatric big data repository	Tabular	Demographic variables and 54 clinical variables	Binary	Obesity	Individual
Park et al [32], 2021	Corpus of geotagged tweets	Text	Tweets	Binary and continuous	BMI and obesity	Individual
Rashmi et al [31], 2021	Objective measure	Image	600 thermograms	Binary	Obesity	Individual
Snekhalatha and Sangamithirai [30], 2021	Objective measure	Image	Thermal imaging	Binary	Diagnosis of obesity	Individual
Thamrin et al [29], 2021	Publicly available health data	Tabular	Risk factors for obesity	Binary	Obesity	Individual
Zare et al [28], 2021	BMI panel data set	Tabular	Kindergarten BMI *z* score	Binary	Obesity by grade 4	Individual

^a^WHR: waist-hip ratio.

^b^MRI: magnetic resonance imaging.

^c^WC: waist circumference.

^d^HC: hip circumference.

^e^BFP: body fat percentage.

^f^EHR: electronic health record.

^g^NHANES: National Health and Nutrition Examination Survey.

^h^HULAFE: Hospital Universitari i Politècnic La Fe.

^i^SAT: subcutaneous adipose tissue.

^j^VAT: visceral adipose tissue.

### Main Findings

Table 3 summarizes the estimated effects and main findings of the studies included in the review. Four key findings have emerged.

First, the studies found that ML or DL models were generally effective in detecting clinically meaningful patterns of obesity or relationships between covariates and weight outcomes; for example, ML and DL models were found useful in classifying obesity severity [30,47,52], identifying anthropometric [34] and genetic characteristics of obesity [56], and predicting obesity onset in children [28,53,63]. ML algorithms (eg, random forest [RF] and conditional RF) revealed meaningful relationships between school and neighborhood environments and overweight and obesity [45,58,62]. DL algorithms (eg, convolutional neural network [CNN]) effectively extracted built environment features from satellite images to assess their associations with the local obesity rate [57].

Second, most (18/22, 82%) of the studies comparing AI models with conventional statistical methods reported that the AI models achieved higher prediction accuracy on test data, whereas others (4/22, 18%) found similar model performances; for example, ML and DL models were found to explain a larger proportion of variations in county-level obesity prevalence than conventional statistical approaches [50]. ML models showed flexibility in handling various variable types [36,41] and large-scale data sets [32] and producing robust, generalizable inferences [41,54,64,65] with higher prediction accuracy [61,66]. By contrast, Cheng et al [37] reported that ML algorithms and conventional statistical approaches had similar performance.

Third, some (5/46, 11%) of the studies comparing the performances of different AI models yielded mixed results, reflecting the interdependence between model and data or task; for example, logistic regressions were reported to achieve higher prediction accuracy than DTs, naïve Bayes (NB) [29], and DL [35]. By contrast, Heydari et al [67] found that logistic regressions and DL models performed equally well in solving classification problems. Zhang et al [68] and Ergün [70] reported that data mining and DL techniques outperformed logistic regressions in classification accuracy.

Fourth, newer studies increasingly adopted state-of-the-art DL models to address CV and NLP tasks; for example, chatbots built on NLP models were used to support pediatric obesity treatment [48]. CNN-based CV models were used to construct indicators for the built environment using images from Google Street View [42]. DL-based tools were used to efficiently visualize and analyze abdominal visceral adipose tissue and subcutaneous adipose tissue [38].

**Table 3:** Estimated effects and main findings of the studies included in the review.

Authors, year	Estimated effects of AI^a^ technologies on obesity prevention or treatment	Main findings
Abdel-Aal and Mangoud [26], 1997	Models for WHR^b^ as a continuous variable predict the actual values within an error rate of 7.5% at the 90% confidence limits. Categorical models predict the correct logical value of WHR with an error in only 2 of the 300 evaluation cases. Analytical relationships derived from simple categorical models explain global observations on the total survey population to an accuracy rate as high as 99%. Simple continuous models represented as analytical functions highlight global relationships and trends. There is a strong correlation between WHR and diastolic blood pressure, cholesterol level, and family history of obesity.	Compared with other statistical and neural network approaches, AIM^c^ abductive networks provide a faster and more automated model synthesis.
Positano et al [71], 2008	CV^d^ values in VAT^e^, SAT^f^, and VAT/SAT ratio assessment by the standard algorithm without image inhomogeneities correction were 10.7%, 11.9%, and 17.3%, respectively. Correlation coefficients were r=0.97, r=0.93, and r=0.95, respectively (all *P*<.001). When correction for field inhomogeneities was applied, VAT, SAT, and VAT/SAT ratio CVs became 9.8%, 6.7%, and 13.1%, respectively. Correlation coefficients became r=0.97, *P*<.001 for VAT; r=0.99, *P*<.001 for SAT; and r=0.97, *P*<.001 for VAT/SAT ratio.	The CV between manual and unsupervised analyses was significantly improved by inhomogeneities correction in SAT evaluation. Systematic underestimation of SAT was also corrected. A less critical performance improvement was found in VAT measurement. The compensation of signal inhomogeneities improves the effectiveness of the unsupervised assessment of abdominal fat. Correction of intensity distortions is necessary for SAT evaluation but less significant in VAT measurement.
Ergün [70], 2009	The classification rate of neural networks in obesity is 90.2%, and the classification rate of logistic regression in obesity is 87.8%. After these classifications, in obesity, the BMI is more affected than the divergent arteries.	The classifying performance of a neural network is better than that of logistic regression.
Yang et al [69], 2009	The implemented method achieved the macroaveraged F-measure of 81% for the textual task and 63% for the intuitive task. The microaveraged F-measure showed an average accuracy of 97% for textual annotations and 96% for intuitive annotations.	Text mining may provide an accurate and efficient prediction of disease statuses from clinical discharge summaries.
Zhang et al [68], 2009	Prediction at 8 months’ accuracy is improved very slightly, in this case by using neural networks, whereas for prediction at 2 years, the obtained accuracy is enhanced by >10%, in this case by using Bayesian methods.	SVM^g^ and Bayesian algorithms seem to be the best algorithms for predicting overweight and obesity from the Wirral database. The incorporation of nonlinear interactions could be important in childhood obesity prediction. Data mining techniques are becoming sufficiently well established to offer the medical research community a valid alternative to logistic regression.
Heydari et al [67], 2012	Regarding logistic regression and neural networks, the respective values were 80.2% and 81.2% for correct classification 80.2% and 79.7% for sensitivity, and 81.9% and 83.7% for specificity; the values for the area under the receiver operating characteristic curve were 0.888 and 0.884, respectively, and the values for the kappa statistic were 0.600 and 0.629, respectively. Abdominal thickness, weight, BMI, and HC^h^ were significantly associated with obesity.	Neural networks and logistic regression were good classifiers for obesity detection but were not significantly different with regard to classification.
Kupusinac et al [66], 2014	The predictive accuracy of an ANN^i^ solution is 80.43%. ANN showed higher predictive accuracy ranging from +1.23% to +3.12%.	An ANN is a new approach to predicting BFP^j^ with the same complexity and costs but with higher predictive accuracy.
Shao [65], 2014	Although the 13 body circumference measurements are involved in the real data set, the proposed models can provide better predictions with fewer body circumference measurements. It is much more convenient to predict BFP with fewer body circumference measurements for most people.	Compared with traditional single-stage approaches, the proposed hybrid models—multiple regression, ANN, multivariate adaptive regression splines, and support vector regression techniques—can effectively predict BFP.
Chen et al [64], 2015	The most important correlated indexes are creatinine, hemoglobin, hematocrit, uric acid, red blood cells, high-density lipoprotein, alanine transaminase, triglyceride, and γ-glutamyl transpeptidase.	The ELM^k^ performs much more efficiently than the SVM and BPNN^l^ and with higher recognition rates. The proposed ELM-based approach for overweight detection in biomedical applications holds promise as a new, accurate method for identifying participants’ overweight status. It provides a viable alternative to traditional overweight modeling tools by offering excellent predictive ability.
Dugan et al [63], 2015	The ID3^m^ model trained on the CHICA^n^ data set demonstrated the best overall performance with an accuracy of 85% and sensitivity of 89%. In addition, the ID3 model had a positive predictive value of 84% and a negative predictive value of 88%. Being overweight between the ages of 12 and 24 months is a key risk factor for obesity after the second birthday. Furthermore, it is more of a risk factor if the child was not overweight before 12 months.	Data from a production clinical decision support system can be used to build an accurate ML^o^ model to predict obesity in children after the age of 2 years.
Nau et al [62], 2015	After examining 44 community characteristics, the researchers identified 13 features of the social, food, and physical activity environment that, in combination, correctly classified 67% of communities as obesoprotective or obesogenic using the mean BMI z score as a surrogate. Social environment characteristics emerged as the most critical classifiers and might leverage intervention.	CRF^p^ allows consideration of the neighborhood as a system of risk factors.
Almeida et al [61], 2016	All BFP-grade predictive models presented a good global accuracy (≥91.3%) for obesity discrimination. Both overfat and obese as well as obese prediction models showed, respectively, good sensitivity (78.6% and 71%), specificity (98% and 99.2%), and reliability for positive or negative test results (≥82% and ≥96%). For boys, the order of parameters, by relative weight in the predictive model, was BMI z score, height, WHtR^q^ squared variable (_Q), age, weight, CC^r^_Q, and HC^s^_Q (adjusted R^2^=0.847 and RMSE^t^=2.852); for girls, it was BMI z score, WHtR_Q, height, age, HC_Q, and CC_Q (adjusted R^2^=0.872 and RMSE=2.171).	BFP can be graded and predicted with relative accuracy from anthropometric measurements (excluding skinfold thickness). Fitness and cross-validation results showed that the multivariable regression model performed better in this population than in some previously published models.
Lingren et al [60], 2016	Overall, the rule-based algorithm performed the best: 0.895 (CCHMC^u^) and 0.770 (BCH^v^).	The rule-based exclusion algorithm performed better than the ML algorithm. The best feature set for ML used Unified Medical Language System concept unique identifiers; International Classification of Diseases, Ninth Revision, codes; and R^x^Norm codes.
Seyednasrollah et al [59], 2017	Replication in the BHSw confirmed the researchers’ findings that WGRSx19 and WGRS97 are associated with BMI. WGRS19 improved the accuracy of predicting adulthood obesity in the training data (area under the curve=0.787 vs area under the curve=0.744; *P*<.001) and validation data (area under the curve=0.769 vs area under the curve=0.747; *P*=.03). WGRS97 improved the accuracy in the training data (area under the curve=0.782 vs area under the curve=0.744; *P*<.001) but not in the validation data (area under the curve=0.749 vs area under the curve=0.747; *P*=.79). Higher WGRS19 is associated with a higher BMI at 9 years and WGRS97 at 6 years.	WGRS19 improves the prediction of adulthood obesity. The model helps screen children with a high risk of developing obesity. Predictive accuracy is highest among young children (aged 3-6 years), whereas among older children (aged 9-18 years), the risk can be identified using childhood clinical factors.
Hinojosa et al [58], 2018	Violent crime, English learners, socioeconomic disadvantage, fewer physical education and fully credentialed teachers, and diversity index were positively associated with obesity. By contrast, the academic performance index, physical education participation, mean educational attainment, and per capita income were negatively associated with obesity. The most highly ranked built or physical environment variables were distance to the nearest highway and green spaces, 10th and 11th most important, respectively.	An RFy algorithm effectively identifies the relative importance of school environment attributes.
Maharana and Nsoesie [57], 2018	Features of the built environment explained 64.8% (RMSE=4.3) of the variation in obesity prevalence across all US census tracts. Individually, the variation explained was 55.8% (RMSE=3.2) for Seattle, Washington (213 census tracts); 56.1% (RMSE=4.2) for Los Angeles, California (993 census tracts); 73.3% (RMSE=4.5) for Memphis, Tennessee (178 census tracts); and 61.5% (RMSE=3.5) for San Antonio, Texas (311 census tracts).	CNN^z^ can be used to automate the extraction of features of the built environment from satellite images for studying health indicators. Understanding the association between specific features of the built environment and obesity prevalence can lead to structural changes that could encourage physical activity and decrease obesity prevalence.
Wang et al [56], 2018	The SVM model significantly outperformed other classifiers based on the same training features. The SVM model exhibits 70.77% accuracy, 80.09% sensitivity, and 63.02% specificity. The selected SNPs^aa^ were effective in the detection of obesity risk.	The ML-based method provides a feasible means for conducting preliminary analyses of genetic characteristics of obesity.
Duran et al [55], 2018	In female participants, the sensitivity of the BMI, WC^bb^, and ANN approaches to predict excess body fat was 0.751 (95% CI 0.730‐0.771), 0.523 (95% CI 0.487‐0.559), and 0.782 (95% CI 0.754‐0.810), respectively. In male participants, the sensitivity of the BMI, WC, and ANN approaches to predict excess body fat was 0.721 (95% CI 0.699‐0.743), 0.572 (95% CI 0.549‐0.594), and 0.795 (95% CI 0.768‐0.821).	The diagnostic performance in identifying excess body fat was better in male participants when an ANN approach was used than when BMI and WC z scores were applied. The ANN and BMI z scores performed comparably and significantly better, respectively, than WC z scores in female participants.
Gerl et al [54], 2019	The lipidome, based on a LASSO^cc^ model, predicted BFP the best (R^2^=0.73). In this model, the strongest positive predictor and strongest negative predictor were sphingomyelin molecules, which differ by only 1 double bond, implying the involvement of an unknown desaturase in obesity-related aberrations of lipid metabolism. The regression was used to probe the clinically relevant information in the plasma lipidome and found that the plasma lipidome also includes information on body fat distribution because WHR (R^2^=0.65) was predicted more accurately than BMI (R^2^=0.47).	ML can model and validate obesity estimates better than classical clinical parameters such as total triglycerides and cholesterol.
Hammond et al [53], 2019	LASSO regression predicted obesity with an area under the receiver operating characteristic curve of 81.7% for girls and 76.1% for boys. In each of the separate models for boys and girls, the researchers found that the weight-for-length z score, BMI between 19 and 24 months, and the last BMI measure recorded before the age of 2 years were the most important features for prediction.	Comparable to cohort-based studies, EHR^dd^ data with area under the receiver operating characteristic curve values could be used to predict obesity at the age of 5 years, reducing the need for investment in additional data collection.
Hong et al [52], 2019	As the results of the 4 ML classifiers showed, the RF algorithm performed the best with micro F1-score 0.9466 and macro F1-score 0.7887 and micro F1-score 0.9536 and macro F1-score 0.6524 for intuitive classification (reflecting medical professionals’ judgments) and textual classification (reflecting the decisions based on explicitly reported information of diseases), respectively. The MIMIC^ee^-III obesity data set was successfully integrated for prediction with minimal configuration of the NLP^ff^2FHIR^gg^ pipeline and ML models.	The FHIR-based EHR phenotyping approach could effectively identify the obesity status and multiple comorbidities using semistructured discharge summaries.
Ramyaa et al [51], 2019	SVM, neural network, and KNN^hh^ algorithms performed modestly for the numerical predictions, with mean approximate errors of 6.70 kg, 6.98 kg, and 6.90 kg, respectively. K-means cluster analysis improved prediction using numerical data and identified 10 clusters suggestive of phenotypes, with a minimum mean approximate error of approximately 1.1 kg. A classifier was used to phenotype participants into the identified clusters, with mean approximate errors of <5 kg for 15% of the test set (approximately, n=2000). SVM performed the best (54.5% accuracy), followed closely by the bagged tree ensemble and KNN algorithms.	SVM regression was the best-suited predictive and inferential tool for this task, closely followed by neural network and KNN algorithms. Although the overall data model showed a good fit and predictive ability, clustering produced relatively superior fit statistics.
Scheinker et al [50], 2019	Multivariate linear regression and gradient boosting machine regression (the best-performing ML model) of obesity prevalence using all county-level demographic, socioeconomic, health care, and environmental factors had R^2^ values of 0.58 and 0.66, respectively (*P*<.001).	ML may be used to explain more variation in county-level obesity prevalence than traditional epidemiologic models. The top-performing ML model explained two-thirds of the variation in county-level obesity prevalence, significantly more than conventional multivariate linear models.
Shin et al [49], 2019	The performance of the proposed system was compared with those of 2 commercial systems that were designed to measure body composition using either a whole body or upper body impedance value. The results showed that the correlation coefficient (R^2^) value was improved by approximately 9%, and the SE of the estimate was reduced by 28%.	The test results validated that the inclusion of anthropometric data helped to improve accuracy, primarily when a DL^ii^ approach was used to predict the regression values.
Stephens et al [48], 2019	Adolescent patients reported experiencing positive progress toward their goals 81% of the time. The 4123 messages exchanged and patients’ reported usefulness ratings (96% of the time) illustrate that adolescents engaged with the chatbot and viewed it as helpful.	An AI chatbot is feasible as an adjunct to treatment. The feasibility and benefit of support through AI, specifically in a pediatric setting, could be scaled to serve larger groups of patients.
Blanes-Selva et al [47], 2020	The PU^jj^ learning algorithm presented a high sensitivity (98%) and predicted that approximately 18% of the patients without a diagnosis were obese.	The implementation of the PU learning methodology in identifying obesity produced results that were satisfactory, providing high sensitivity, and consistent with the World Health Organization’s obesity report.
Dunstan et al [46], 2020	Using only 5 categories, RF could predict obesity prevalence with absolute error <10% for approximately 60% of the countries considered and absolute error <20% for 87%. The most relevant food category with regard to predicting obesity consists of baked goods and flours, followed by cheese and carbonated drinks.	RF shows the best performance for predicting obesity from food, followed closely by XGB^kk^.
Fu et al [45], 2020	The 2 most important features—trajectory of infant BMI z score change and maternal BMI at enrollment—were identified from the ML algorithm. The aforementioned features showed similar predictive capacity compared with all features (area under the curve=0.68 vs 0.68; *P*=.83; DeLong test). The sensitivity analyses identified the same 2 features (ie, trajectory of infant BMI z score change and maternal BMI at enrollment), and the ranking of these features’ Shapley additive explanations value was unchanged. In the independent test cohort, the area under the curve for childhood overweight and obesity classification using the aforementioned 2 features was 0.71 (95% CI 0.66 to 0.76), which was comparable to that based on all features (0.72, 95% CI 0.67 to 0.76).	An ML algorithm is applied to identify risk factors contributing to childhood overweight or obesity based on a large longitudinal study and addresses the relationships between all collected features and outcomes without any assumption. A novel unified framework, Shapley additive explanations, is used to interpret predictions, and the identified predictive factors are robust.
Kibble et al [44], 2020	New potential links between cytokines and weight gain are identified, as well as associations among dietary, inflammatory, and epigenetic factors.	An integrative ML method called group factor analysis was used to identify the links between multimolecular-level interactions and the development of obesity.
Park et al [43], 2020	The actual and predicted ΔBMI showed a significant intraclass correlation value with a low RMSE, and classification between people with increased BMI and those with nonincreased BMI resulted in a high area under the receiver operating characteristic curve value using only the degree centrality values obtained at the baseline visit.	The constructed model using functional connectivity of the selected regions provides robust neuroimaging biomarkers for predicting BMI progression.
Phan et al [42], 2020	A DNN^ll^ was used for neighborhood indicator recognition and achieved high accuracies (85%-93%) for the separate recognition tasks.	DL techniques were used to create indicators for neighborhood-built environment characteristics.
Taghiyev [41], 2020	The proposed hybrid system demonstrated 91.4% accuracy, which is higher than that of other classifiers (ie, 4.6% higher than the performance of logistic regression and 2.3% higher than the performance of DT^mm^).	The proposed hybrid system provides a more accurate classification of patients with obesity and a practical approach to estimating the factors affecting obesity.
Xiao et al [40], 2020	All aspects of horizontal greenery, vertical greenery, and proximity of green levels affected body weight; however, only the VGI^nn^ consistently had an adverse effect on weight and obesity.	The VGI of the DL approach using Baidu Street View images could effectively capture the eye-level greenness in high-density–population areas. Thus, VGI can be used to effectively promote walking and other physical activities to prevent obesity.
Yao et al [39], 2020	Jogging may be a more suitable activity of daily living for BMI prediction than walking and walking up stairs.	The proposed DL model with the motion entropy–based filtering strategy outperforms the baseline approaches significantly.
Alkutbe et al [27], 2021	For the gradient boosting models, the predicted fat percentage values were more aligned with the actual value than those in regression models. Gradient boosting achieved better performance than the regression equation because it combined multiple simple models into a single composite model to take advantage of this weak classifier. The developed predictive model archived RMSE values of 3.12 for girls and 2.48 for boys.	ML models and newly developed centile charts could be valuable tools for estimating and classifying BFP.
Bhanu et al [38], 2021	The accuracy of segmentation was superficial SAT: 0.92, deep SAT: 0.88, and VAT: 0.9. The average Hausdorf distance was <5 mm. Automated segmentation significantly correlated R^2^>0.99 (*P*<.001) with ground truth for all 3-fat compartments. Predicted volumes were within 1.96 SD from Bland-Altman analysis.	DL-based, comprehensive superficial SAT, deep SAT, and VAT analysis tools showed high accuracy and reproducibility and provided a comprehensive fat compartment composition analysis and visualization in <10 seconds.
Cheng et al [37], 2021	Physical activity was an important factor in predicting weight status, with gender, age, and race or ethnicity being less important factors associated with weight outcomes. The durations of vigorous-intensity activity in 1 week and moderate-intensity activity in 1 week were essential attributes.	With physical activity and basic demographic information of all methods analyzed, the random subspace classifier algorithm achieved the highest overall accuracy and area under the receiver operating characteristic curve value. In general, most algorithms showed similar performance. Logistic regression was middle ranking in terms of overall accuracy, sensitivity, specificity, and area under the receiver operating characteristic curve value among all methods.
Delnevo et al [36], 2021	The psychological variables in use allow one to predict both BMI values (with a mean absolute error of 5.27-5.50) and BMI status with an accuracy of >80% (metric: F1-score).	Certain psychological variables such as depression are highly predictive of BMI. ML has several advantages over traditional statistics and can be used to compare the impact of many variables on predicting a chosen outcome and can handle various types of variables.
Lee et al [35], 2021	For predicting a newborn’s BMI, linear regression (2.0744) and RF (2.1610) were better than ANN with 1, 2, and 3 hidden layers (150.7100, 154.7198, and 152.5843, respectively) in the mean squared error. On the basis of variable importance from the RF, the major predictors of a newborn’s BMI were the first abdominal circumference value and estimated fetal weight in week 36 or later, gestational age at delivery, the first abdominal circumference value during week 21 to week 35, maternal BMI at delivery, maternal weight at delivery, and the first biparietal diameter value in week 36 or later.	ML approaches based on ultrasound measures would be a useful noninvasive tool for predicting a newborn’s BMI. Linear regression and RF were better models than ANNs for predicting a newborn’s BMI.
Lin et al [34], 2021	ML revealed the following 4 stable metabolically distinct obesity clusters in each cohort: Metabolic healthy obesity (44% of the patients) was characterized by a relatively healthy metabolic status with the lowest incidents of comorbidity. Hypermetabolic obesity–hyperuricemia (33% of the patients) was characterized by extremely high uric acid and an increased incidence of hyperuricemia (adjusted odds ratio 73.67 to metabolic healthy obesity, 95% CI 35.46-153.06). Hypermetabolic obesity–hyperinsulinemia (8% of the patients) was distinguished by overcompensated insulin secretion and an increased incidence of polycystic ovary syndrome (adjusted odds ratio 14.44 to metabolic healthy obesity, 95% CI 1.75-118.99). Hypometabolic obesity (15% of the patients) was characterized by extremely high glucose levels, decompensated insulin secretion, and the worst glucolipid metabolism (diabetes: adjusted odds ratio 105.85 to metabolic healthy obesity, 95% CI 42.00-266.74; metabolic syndrome: adjusted odds ratio 13.50 to metabolic healthy obesity, 95% CI 7.34-24.83). The assignment of patients in the verification cohorts to the main model showed a mean accuracy of 0.941 in all clusters.	ML automatically identified 4 subtypes of obesity in clinical characteristics in 4 independent patient cohorts. This proof-of-concept study provided evidence that a precise diagnosis of obesity can potentially guide therapeutic planning and decisions for different subtypes of obesity.
Pang et al [33], 2021	XGB yielded a mean area under the curve value of 0.81 (SD 0.001), which outperformed all other models. It also achieved a statistically significant better performance than all other models on standard classifier metrics (sensitivity fixed at 80%): precision, mean 30.9% (SD 0.22%); F1-score, mean 44.6% (SD 0.26%); accuracy, mean 66.14% (SD 0.41%); and specificity, mean 63.27% (SD 0.41%).	The presented ML model development workflow can be adapted to various EHR-based studies and is valuable for developing other clinical prediction models.
Park et al [32], 2021	ML algorithms were used to determine the stances of tweets on Black Lives Matter. ML models showed better performance than lexicon-based sentiment analysis (accuracy: 61%). The NB^oo^ model had an overall accuracy of 85%, slightly higher than that of the CNN model (83.8%); both had higher accuracy than the other models. However, NB had the highest recall and F1-score for predicting the against stance, whereas CNN performed poorly on identifying the against stance.	The study demonstrated the strengths of ML techniques in handling large data sets. Social scientists can use ML techniques to scale up traditional content analysis.
Rashmi et al [31], 2021	The PCA^pp^ method provides the best classification accuracy for SVM (98%), followed by NB and RF (97%).	The regional thermography and computer-aided diagnostic tool with ML classifier could be used as a primary noninvasive prognostic tool for evaluating obesity in children.
Snekhalatha and Sangamithirai [30], 2021	Among the region of interest studied, the abdomen region exhibited a high temperature difference of 4.703% between normal participants and participants who were obese compared with other regions. The proposed custom network-2 provided an overall accuracy of 92%, with an area under the curve value of 0.948. By contrast, the pretrained model VGG16 produced an accuracy of 79% and an area under the curve value of 0.90 for discrimination into obese and normal thermograms.	The DL system based on custom CNN provided a reliable classification performance to identify the occurrence of obesity in test participants. Custom CNN network-2 provided a commendable accuracy in classifying normal participants and participants who were obese from the thermal images. The trained custom-2 CNN model can be used for computer-aided screening of test participants for obesity detection.
Thamrin et al [29], 2021	Location, marital status, age group, education, sweet drinks, fatty or oily foods, grilled foods, preserved foods, seasoning powders, soft drinks or carbonated beverages, alcoholic beverages, mental or emotional disorders, diagnosed hypertension, physical activity, smoking, and fruit and vegetable consumption are significant in predicting obesity status in adults. The classification prediction using the logistic regression method achieves the best performance based on the accuracy metric (72%), specificity (71%), precision (69%), kappa (44%), and Fβ-score (70%). Classification prediction by the classification and regression tree method achieves the highest sensitivity (82%) and the highest F1-score (72%). With regard to the area under the receiver operating characteristic curve performance of the respective classification methods with 10-fold cross-validation, the logistic regression classifier has the highest average area under the receiver operating characteristic curve value (0.798).	Logistic regression has a better performance than the classification and regression tree and NB methods. Kappa coefficients show only moderate concordance between predicted and measured obesity. The constructed obesity classification model can evaluate and predict the risk of obesity using ML methods for the population of Indonesia, which can then be applied to publicly available open data.
Zare et al [28], 2021	The kindergarten BMI z score is the most important predictor of obesity by grade 4. Including the kindergarten BMI z score of students in the model meaningfully increases the prediction accuracy. Logistic regression, RF, and neural network algorithms performed similarly in terms of accuracy, sensitivity, specificity, and area under the curve values. The 95% CIs around the area under the curve overlap among these 3 algorithms. The DT showed lower performance with an area under the curve value that was statistically lower than the area under the curve values from each of the other algorithms. Nevertheless, the performance of the DT algorithm was close to that of the others.	Data from the Arkansas, United States, BMI screening program significantly improve the ability to identify children at a high risk of obesity to the extent that better prediction can be translated into more effective policy and better health outcomes. The ability to predict obesity by grade 4 was robust across the ML algorithms and logistic regression with these data.

^a^AI: artificial intelligence.

^b^WHR: waist-to-hip ratio.

^c^AIM: abductory induction mechanism.

^d^CV: coefficient of variation.

^e^VAT: visceral adipose tissue.

^f^SAT: subcutaneous adipose tissue.

^g^SVM: support vector machine.

^h^HC: hip circumference.

^i^ANN: artificial neural network.

^j^BFP: body fat percentage.

^k^ELM: extreme learning machine.

^l^BPNN: back propagation neural network.

^m^ID3: iterative dichotomizer 3.

^n^CHICA: Child Health Improvement Through Computer Automation.

^o^ML: machine learning.

^p^CRF: conditional random forest.

^q^WHtR: waist-to-height ratio.

^r^CC: calf circumference.

^s^HC: hip circumference.

^t^RMSE: root mean square error.

^u^CCHMC: Cincinnati Children’s Hospital and Medical Center.

^v^BCH: Boston Children’s Hospital.

^w^BHS: Bogalusa Heart Study.

^x^WGRS: weighted genetic risk score.

^y^RF: random forest.

^z^CNN: convolutional neural network.

^aa^SNP: single-nucleotide polymorphism.

^bb^WC: waist circumference.

^cc^LASSO: least absolute shrinkage and selection operator.

^dd^EHR: electronic health record.

^ee^MIMIC: Multiparameter Intelligent Monitoring in Intensive Care.

^ff^NLP: natural language processing.

^gg^FHIR: Fast Healthcare Interoperability Resources.

^hh^KNN: k-nearest neighbor.

^ii^DL: deep learning.

^jj^PU: positive and unlabeled.

^kk^XGB: extreme gradient boosting.

^ll^DNN: deep neural network.

^mm^DT: decision tree.

^nn^VGI: Visible Green Index.

^oo^NB: naïve Bayes.

^pp^PCA: principal component analysis.

### Methodological Review

#### AI Overview

AI symbolizes the effort to automate intellectual tasks usually performed by humans [72]. In general, AI consists of 2 domains or developmental periods: symbolic AI and modern AI [73]. Symbolic AI prevailed from the 1950s to the 1980s, characterized by the endeavors to achieve human-level intelligence by having programmers handcraft a sufficiently large set of explicit rules for manipulating knowledge [74]. Although symbolic AI proved suitable for solving well-defined, logical problems, such as a rule-based question-answer system, it became intractable when creating rules to solve more complex, fuzzy issues such as image classification, speech recognition, and language translation [74]. The definition of ML is “the field of study that gives computers the ability to learn without being explicitly programmed” [75]. Instead of hard coding all the rules in the symbolic AI, researchers provide examples (eg, images with labels that identify the objects in them) to *train* modern ML models to output rules [74]. As a subdomain of ML, DL is based on artificial neural networks in which multiple (*deep*) layers of artificial neurons are used to progressively extract higher-level features from data [76]. This layered representation enables the modeling of more complex, dynamic patterns compared with traditional ML (which sometimes is called *shallow learning* in contrast to DL), which finds its utility in analyzing *big data*: data massive in scale and *messy* to work with (eg, unstructured texts and images) [77]. The first ML and DL algorithms were developed in the 1950s, attracting initial excitement but then lying dormant for several decades [72]. Since the late 1980s, partly because of the rediscovery of backpropagation algorithms, the invention of CNNs, and the strong growth in computational capacity, ML and DL have regained their popularity vis-à-vis symbolic AI [72].

#### AI Versus Conventional Statistical Methods

Admittedly, the concept of conventional statistical methods is dubious at best because the development of statistical theories and algorithms is continual in time and intertwines at all levels [78]. Indeed, many *conventional* models fall into the ML domain, such as linear and logistic regressions. Despite the poorly defined domain and overlapping algorithms, at least 2 distinctions could be made between modern AI (ie, ML and DL) and other statistical methods. In terms of aims, the objective of AI models and their evaluation metrics predominantly concern prediction precision (often at the cost of compromising interpretability as models become complex) [78,79]. By contrast, conventional statistical approaches usually attempt to reveal relationships among variables (*statistical inference*) and focus on model interpretability [80]. In terms of procedures, it is standard practice to split data into training, validation, and test sets so that an AI model can be trained using the training set with the aim of achieving the optimal performance on some predefined evaluation metrics (eg, accuracy and mean squared error) when testing on the validation set [81,82]. The fine-tuned AI model is subsequently tested on the test set. The utility of the validation set is to prevent model overfitting (ie, too tailored to the training set while losing generalizability to new, unseen data) and fine-tune hyperparameters (ie, parameters external to the model, whose values cannot be automatically learned from data). The test set is preserved to test the final model’s performance on unseen data. By contrast, conventional statistical methods do not usually fit and evaluate models using training, validation, and test sets but use other model selection criteria (eg, adjusted R-squared and Akaike and Bayesian information criteria) to evaluate model performance [83].

#### ML Subcategories

##### Overview

ML is classified into 2 subcategories: unsupervised ML and supervised ML [84]. Unsupervised ML analyzes and clusters unlabeled data sets, discovering hidden patterns or data groupings without the need for human intervention [85]. Its capability to reveal similarities and differences in information makes it ideal for exploratory data analysis. Unsupervised ML models are used for 3 main tasks: clustering, association, and dimensionality reduction [86]. Clustering algorithms (eg, k-means clustering, hierarchical clustering, and Gaussian mixture) group unlabeled data based on similarities [86]. Association algorithms (eg, Apriori, Eclat, and FP-Growth) identify rules and relations among variables in large databases [87]. Dimensionality reduction algorithms (eg, principal component analysis [PCA], singular value decomposition, and multidimensional scaling) deal with an excessive number of features during data preprocessing, reducing them to a manageable size while preserving the integrity of the data set as much as possible [88]. Supervised ML uses a training set consisting of input-output pairs to enable the algorithm to learn a function that maps input to output over time [89]. The algorithm measures its accuracy through the loss function, adjusting until the error is minimized sufficiently. The critical difference between supervised ML and unsupervised ML is that the former requires labeled data (ie, input-output pairs), whereas the latter only requires inputs (ie, unlabeled data) [84]. Supervised ML models are used for 2 main tasks: classification and regression [84]. Classification algorithms assign data to specific categories (eg, obese or nonobese). Regression algorithms learn the relationship between input features and continuously distributed outcomes and are commonly used for projections (eg, BMI in 5 years).

##### Unsupervised ML

###### K-means Clustering

K-means clustering is an iterative algorithm that tries to partition the data set into a total of *k* nonoverlapping groups (ie, clusters) [86,90]. Each data point belongs to only 1 group. The algorithm attempts to make the intracluster data points as similar as possible while keeping the clusters apart. In particular, it assigns data points to a cluster such that the sum of the squared distance between the data points and the cluster’s centroid (ie, arithmetic mean of all the data points belonging to that cluster) is minimized. As the number of clusters *k* needs to be determined before implementing the algorithm, silhouette coefficients are commonly used to identify the optimal *k* value. Lin et al [34] used k-means clustering to classify patients with obesity into 4 groups based on 3 biomarkers concerning glucose, insulin, and uric acid.

###### Fuzzy C-means Clustering

In nonfuzzy clustering (also known as hard clustering; for example, k-means clustering), data are divided into distinct clusters, where each data point can only belong to 1 cluster [86]. In fuzzy clustering, data points can potentially belong to multiple clusters [91]. Fuzzy c-means clustering assigns each data point membership from 0% to 100% in each cluster center [92]. The fuzzy partition coefficient is often used to determine the optimal number of clusters with a value ranging from 0 (worst) to 1 (best) [93]. Positano et al [71] used the fuzzy c-means algorithm to classify MRI pixels into clusters to assess abdominal fat.

###### Group Factor Analysis

Factor analysis describes relationships among the individual variables of a data set [94]. Group factor analysis (GFA) extends this classical formulation into describing relationships among groups of variables, where each group represents either a set of related variables or a data set [95]. GFA is commonly formulated as a latent variable model consisting of 2 hierarchical levels: the higher level models the relationships among the groups, and the lower-level models the observed variables given the higher level [95]. Kibble et al [44] used GFA to jointly analyze 5 large multivariate data sets to understand the multimolecular-level interactions associated with obesity development.

###### PCA for Large Data Sets

Large data sets are increasingly common nowadays. PCA is a classic, widely adopted method to reduce the dimensionality of a large data set while preserving as much statistical information (ie, variability) as possible [86]. In particular, PCA attempts to find new variables, called principal components, that are linear functions of those in the original data set. The new variables are uncorrelated with each other (ie, orthogonal) and maximize the projected data variance. Rashmi et al [31] used PCA to reduce the feature dimensions of a thermal imaging data set to classify children by their obesity severity level.

##### Supervised ML

###### Linear Regression

Linear regression is considered a conventional statistical model and a classical architecture to develop a predictive model [96], but it fulfills all criteria from an ML point of view and is widely used as an ML algorithm to predict continuous outcomes such as BMI or BFP [97]. Trainable weights (ie, coefficients) of linear regression are commonly estimated using ordinary least squares or gradient descent. Compared with many other ML models, linear regression has the advantages of simplicity and interpretability [98]. It is easy to understand how the model reaches its predictions. Wang et al [56] used linear regressions to identify features of single-nucleotide polymorphisms that predict obesity risk. Phan et al [42] used linear regressions to estimate the associations between built environment indicators and state-level obesity prevalence.

###### Regularized Linear Regression

The bias-variance tradeoff is a fundamental issue faced by all ML models [86,99]. Bias is an error from erroneous assumptions in a learning algorithm. High bias may cause the algorithm to miss the relevant relations between features and outputs (called underfitting). Variance is an error from a learning algorithm’s sensitivity to small fluctuations in the training set. A high variance may result from the algorithm modeling the random noise in the training data, often leading to the algorithm’s poor generalizability to new, unseen data (called overfitting). In general, decreasing variance increases bias and vice versa, and ML algorithms need to be fine-tuned to balance these 2 properties. Regularization is an essential technique to prevent model overfitting and improve generalizability (at the cost of increasing bias) by adding a penalty term of trainable weights to the loss function [86]. Optimization algorithms that minimize the loss function will learn to avoid extreme weight values and thus reduce variance. The penalty term with the sum of squared trainable weights is called L2 regularization, used in Ridge regression. The penalty term with the sum of the absolute values of trainable weights is called L1 regularization, used in the least absolute shrinkage and selection operator (LASSO) regression. Unlike Ridge regression, LASSO regression often shrinks some feature weights to absolute zero, making it useful for feature selection. Finally, ElasticNet regression uses a weighted sum of L1 and L2 regularizations. Gerl et al [54] used LASSO regression to estimate the relationship between human plasma lipidomes and body weight outcomes, including BMI, WC, WHR, and BFP.

###### Logistic Regression

In its simplest form, logistic regression uses a logistic function, called the sigmoid function, to model a binary outcome [100]. A sigmoid function is a continuous, smooth, differentiable S-shaped mathematical function that maps a real number to a value in the range of 0 and 1, making it ideal for modeling probabilities. The estimated probabilities are converted to predictions (0 or 1, denoting exclusive group membership) based on some predefined threshold (eg, >0.5). In ML, logistic regression often incorporates regularizations (L1, L2, or both) to prevent overfitting. Another common extension of logistic regression in ML is to solve multiclass classification problems when classification tasks involve >2 (exclusive) classes. A typical strategy uses the one-vs-rest method (also called one-vs-all) that fits 1 classifier (eg, a logistic regression) per class against all the other classes [101]. A data point is assigned to the class with the highest confidence score among all classifiers. Thamrin et al [29] used logistic regressions to assess the predictability of various obesity risk factors. Cheng et al [37] used logistic regressions to classify obesity status based on participants’ physical activity levels.

###### NB Classifier

NB algorithms apply the Bayes theorem with the *naïve* assumption of conditional independence among each pair of features given the value of the class [102]. Despite this oversimplified assumption, NB classifiers have been widely used and have worked well in solving many real-world problems. The decoupling of conditional feature distributions allows each distribution to be independently estimated as 1D, making the training of NB classifiers much faster than more sophisticated ML models [86]. By contrast, the predicted probabilities of NB classifiers are less trustworthy owing to the algorithm’s *naïve* assumption. Rashmi et al [31] used NB to classify childhood obesity based on thermogram images. Thamrin et al [29] adopted NB to predict adult obesity using Indonesian health survey data [29].

###### K-nearest Neighbor

K-nearest neighbor (KNN) is a nonparametric, supervised learning algorithm suitable for classification and regression tasks [103]. The input consists of the *k* closest training data points based on a prespecified distance measure (eg, Euclidean, Manhattan, or Minkowski distance). For classification tasks, the output is a class membership. A test data point is assigned to the class most common among its k-nearest neighbors (if *k*=1, the test data point is assigned to the class of the single nearest neighbor). For regression tasks, the output is the average value of its k-nearest neighbors. KNN should not be confused with k-means. The former is a supervised ML algorithm to determine the class or value of a data point based on its k-nearest neighbors, whereas the latter is an unsupervised ML algorithm to classify data points into *k* clusters that minimize the distances within clusters while maximizing those between clusters [90]. KNN is a memory-based learning algorithm that requires no training (called a lazy learner) but can become significantly slower when the sample size increases. Wang et al [56] used KNN to predict obesity risk based on features of single-nucleotide polymorphisms. Ramyaa et al [51] performed KNN to predict body weight using physical activity and dietary data.

###### Support Vector Machines

Support vector machines (SVMs), which are supervised learning models that construct a hyperplane in a high-dimensional space, can be used for classification and regression tasks [104]. SVMs attempt to identify the hyperplane separating different classes while maximizing the distance to any class’s nearest training data point (ie, margin). Intuitively, the larger the margin, the more likely the model’s generalizability to new, unseen data. The choice of margin type can be critical for SVMs [86]. Hard-margin SVMs maximize the margin by minimizing the distance from the decision boundary to the training points. However, hard-margin SVMs may lead to overfitting and have no solution if the training data are linearly inseparable. Soft-margin SVMs modify the constraints of the hard-margin SVMs by allowing some data points to violate the margin (ie, misclassified). In practice, data are seldom linearly separable in the original feature space, and kernel methods are applied to map the input space of the data to a higher-dimensional feature space where linear models can be trained [105]. Many kernel functions, such as the Gaussian radial basis, sigmoid, and polynomial kernel, can be chosen. Wang et al [56] used SVM to predict obesity risk based on the features of single-nucleotide polymorphisms. Ramyaa et al [51] applied SVM to predict body weight using physical activity and diet data.

###### DT Algorithms

DTs are nonparametric supervised learning methods for classification and regression tasks [106]. In DT algorithms, a tree is built by splitting the source set that constitutes the tree’s root node into subsets, which comprise the successor children [107]. The splitting is based on a set of rules applied to input features. Different splitting rules exist, such as variance reduction for regression tasks and Gini impurity or information gain for classification tasks. The splitting process is repeated on each derived subset recursively (ie, recursive partitioning). The recursion is completed when all subsets at a node share the same target value or when splitting no longer adds value to the predictions. DTs have several advantages over other ML algorithms, such as high transparency and interpretability and few requirements for data preprocessing [108]. However, DTs can be prone to overfitting (ie, too confident about the rules learned from the training set, which does not generalize well to the test set) and instability (minor variations in the data resulting in a very different tree). Using features extracted from electronic medical records, Hong et al [52] used DTs to predict obesity and 15 other comorbidities. Taghiyev et al [41] performed DTs to identify risk factors associated with obesity onset.

###### RF Models

Ensemble methods are approaches that aggregate the predictions of a group of models aiming for improved performance in classification or regression tasks [109]. Various ensemble methods exist, such as bagging, pasting, boosting, and stacking [86]. Bagging and pasting use the same training algorithm for every predictor included in the ensemble and train it on different random subsets of the training set. When sampling is performed with replacement, the method is called bagging; when sampling is performed without replacement, it is called pasting. RF is an ensemble of DTs commonly trained via the bagging or pasting method [110]. Specifically, RF fits many DTs on various subsets of the data and uses averaging to improve the predictive accuracy and prevent overfitting. For classification tasks, the RF output is the class selected by most trees; for regression tasks, the mean prediction of the individual trees is used. Some common hyperparameters of RF for fine-tuning include the number of trees in the forest, the maximum number of features considered for splitting a node, the maximum number of branches in each tree, the minimum number of data points placed in a node before the node is split, the minimum number of data points allowed in a leaf node, and the method for sampling data points (ie, with or without replacement) [86]. RF typically produces more accurate and robust predictions than DTs and is one of the most popular supervised ML algorithms [111]. Using RF models, Hinojosa et al [58] examined the relationship between social and physical school environments and childhood obesity in California, United States. Dunstan et al [46] performed RF to predict national obesity prevalence using food sales data from 79 countries.

###### Extreme Gradient Boosting

Boosting refers to any ensemble method that combines several weak models into a strong one [112]. The difference between boosting and bagging and pasting is that in boosting, different models are applied to the entire training set sequentially, the new model attempting to address the weaknesses (eg, misclassified targets and residual errors) of the previous model. By contrast, in bagging and pasting, the same models are trained on different random subsets of the training set. A popular boosting algorithm is gradient boosting, in which the new model is trained on the residual errors made by the previous model [113]. Extreme gradient boosting (XGBoost) implements an optimized, parallel-tree gradient boosting algorithm, aiming to be highly efficient, flexible, and portable [114]. XGBoost is considered one of the most powerful ML algorithms, often serving as an essential component of winning entries in ML competitions [86]. A few drawbacks of XGBoost include lacking interpretability and being prone to overfitting. Pang et al [33] used XGBoost to predict early childhood obesity based on electronic health records. Alkutbe et al [27] applied gradient boosting to predict BFP based on cross-sectional health survey data collected in Saudi Arabia.

###### Multivariate Adaptive Regression Splines

Multivariate adaptive regression splines (MARS) is a nonparametric regression technique that automatically models nonlinearities and interactions among variables by combining ≥2 linear regressions using hinge functions [115,116]. A hinge function is a function equal to its argument where that argument is >0 and 0 everywhere else. MARS builds a model using a 2-phase procedure [117]. The forward phase starts with a model consisting of only the intercept term (ie, mean of the target) and repeatedly adds basis functions (ie, constant or hinge function) in pairs to the model that minimizes the squared error loss of the training set. The backward (or pruning) phase usually starts with an overfitted model and removes its least effective term at each step until the best submodel is found. MARS requires little or no data preparation, is easy to understand and interpret, and can address classification and regression tasks. However, it often underperforms boosting ensemble methods. Shao [65] applied MARS to predict BFP using a small-scale health record data set.

#### DL Models

In the obesity literature reviewed, DL models were applied to 3 distinct data types: tabular data (eg, spreadsheet data), images, and texts. The model architectures differ systematically across these data types.

##### DL on Tabular Data

Although *shallow* ML models perform well on tabular data sets in most cases, some complex relationships between the features and the target could be more effectively learned by a deep neural network model [118]. A fully connected neural network consists of a series of fully connected layers, with each artificial neuron (ie, node) of a layer linking with all neurons in the following layer [76]. A multilayer perceptron (MLP) is a classic fully connected neural network consisting of at least 3 layers of neurons: an input layer, a hidden layer, and an output layer [119]. One advantage of fully connected neural networks is that they are *structure agnostic*, requiring no specific assumptions about the input. However, neural networks trained on tabular data can sometimes be prone to overfitting [120]. Park and Edington [121] used MLP to identify individuals at elevated diabetic risk. Heydari et al [67] performed MLP to predict obesity status using data from a cross-sectional study of military personnel in Iran.

##### DL on Images

CV is a field of AI that enables computers to learn from digital images, videos, or other visual inputs and derive meaningful information for decision-making and recommendations [122,123]. Nowadays, most CV applications use DL models, which prove more capable than their *shallow-learning* (ie, ML models) counterparts in representing and revealing high-dimensional, complex nonlinear patterns inherent in image data. Specifically, CNNs consistently outperform the traditional densely connected neural networks (eg, MLP) and achieve human-like or superhuman accuracy in many challenging CV tasks ranging from image classification to object detection and segmentation [124,125]. The main advantages of CNNs over densely connected neural networks are locality, translation invariance, and computational efficiency [126]. Locality refers to the repeated use of small-sized kernels (or filters) in CNNs to identify local patterns at an increasing level of complexity (eg, from basic shapes such as lines and edges to complex objects such as adipose tissue or brain tumor). Translation invariance refers to CNNs’ capacity to detect an entity independent of its position in the image. The computational efficiency of CNNs is achieved by using kernels, global pooling, and other techniques, which typically make the models much smaller (ie, fewer learnable parameters) than their densely connected counterparts. Over the past decade, numerous CNN-based DL models were built and adopted to tackle domain-specific CV problems [76,127]. Some landmark models include, but are not limited to, LeNet, AlexNet, VGG, Inception, ResNet, Xception, ResNeXt, and U-Net.

Transfer learning plays a crucial role in modern AI, where a model developed for a task is reused as the starting point for a model on a different but related task [128]; for instance, the ResNet model trained on ImageNet data with >14 million images in approximately 1000 categories (eg, tables and horses) has stored many useful visual patterns in its weights, which can help solve other CV tasks (eg, identifying fat tissues in MRI scans) [129]. Transfer learning can substantially reduce the number of images required to train a model for a particular task and boost model performance compared with models trained from scratch [130].

Maharana and Nsoesie [57] adopted the VGG model architecture to examine the relationship between obesity prevalence and the built environment measured by Google Maps images (eg, parks, highways, green streets, crosswalks, and diverse housing types). Similarly, Phan et al [42] used the VGG model to assess the link between the statewide prevalence of obesity, physical activity, and chronic disease mortality and the built environment using images from Google Street View. Bhanu et al [38] applied the U-Net model to identify adipose tissues from MRI data. Snekhalatha and Sangamithirai [30] applied transfer learning on a pretrained CNN model to detect obesity based on thermal imaging data.

##### DL on Text

Besides CV, NLP is another field where DL dominates [131]. Early NLP models primarily adopted recurrent neural network (RNN) architecture, demonstrating broad applicability to various NLP tasks such as sentiment analysis, text summarization, language translation, and speech recognition [74,132]. RNN differs from feed-forward MLP in that it takes information from prior inputs (stored as *memories*) to influence the current input and output, which capitalizes on the structure of sequential data where order matters (eg, time series or natural languages) [133]. Some popular RNN models used in NLP tasks include gated recurrent unit and long short-term memory unit [74]. However, in today’s NLP landscape, transformers, invented by a team at Google in 2017, have surpassed RNN models such as gated recurrent unit and long short-term memory unit [134-136]. Transformers are encoder-decoder models that use self-attention to process language sequences [137]. An encoder maps an input sequence into state representation vectors. A decoder decodes the state representation vector to generate the target output sequence. The self-attention mechanism is used repeatedly within the encoder and the decoder to help them contextualize the input data. Specifically, the mechanism compares every word in the sentence to every other word, including itself, and reweighs each word’s embeddings to incorporate contextual relevance. Popular transformer models such as GPT-3, BERT, XLNet, RoBERTa, and T5 have been widely applied to various NLP tasks and achieved state-of-the-art results [137]. Stephens et al [48] tested the efficacy of pediatric obesity treatment support through Tess, a behavioral coaching chatbot built on NLP models. The study concluded that Tess demonstrated therapeutic values to pediatric patients with obesity and prediabetes, especially outside of office hours, and could be scaled up to serve a larger patient population.

## Discussion

### Overview

This study conducted a scoping review of the applications of AI to obesity research. A keyword search in digital bibliographic databases identified 46 studies that used diverse ML and DL models to study obesity-related outcomes. In general, the studies found AI models helpful in detecting clinically meaningful patterns of obesity or relationships between specific covariates and weight outcomes. The majority (18/22, 82%) of the studies comparing AI models with conventional statistical approaches found that the AI models achieved higher prediction accuracy on test data. Some (5/46, 11%) of the studies comparing the performances of different AI models revealed mixed results, likely indicating the high contingency of model performance on the data set and task it was applied to. An accelerating trend of adopting state-of-the-art DL models over standard ML models was observed to address challenging CV and NLP tasks. We concisely introduced the popular ML and DL models and summarized their specific applications in the studies included in the review.

Despite the variety of ML and DL models used in obesity research, it could well be the beginning of the trend for using AI applications in the big data era. Future adoptions of AI in obesity research could be influenced by a broad spectrum of factors, with 3 prominent ones discussed in the following sections.

### Artificial General Intelligence

The ML and DL models reviewed in this study were primarily unimodal and task specific: they were built on a single data type (eg, tabular, text, or image) to solve a specific problem such as obesity classification or BMI prediction. Recent advances in AI showcase the feasibility and possibly superior performance of multimodal, multitask ML and DL models that are trained on diverse data types (eg, tabular plus text, image, video, or audio) and can handle many domains of downstream tasks (eg, text generation, object detection, time series prediction, and speech recognition) simultaneously [138-140]. However, it should be noted that the predictive accuracy of AI models may vary across gender and age groups [27] and sex and age groups [59]. Different from BMI, BMI *z* scores adjust for sex and age differences [141]. Future research may evaluate the potential disparities in AI model performances in their applications to BMI versus BMI *z* scores as outcome measures. Artificial general intelligence (AGI) refers to the ability of an intelligent agent to understand or learn any intellectual task performed by a human being [142,143]. It is too early to tell whether these multimodal, multitask ML and DL models may lead to AGI (or whether we could ever achieve AGI through technological innovations) [144]. Nevertheless, we may soon witness increasing applications of these models in obesity-related research.

### Synthetic Data Generation

Data access is fundamental to any AI model training. Two primary barriers with regard to data are limited sample size and confidentiality concerns [145-148]. ML and DL models are increasingly used to generate synthetic data as an alternative to data collected from the real world [149,150]. Synthetic data do not contain private information requiring human subject review and, therefore, can be shared with other parties or the public without confidentiality concerns [151]. By contrast, synthetic data preserve the original data’s mathematical and statistical properties, ensuring that the AI model trained on them can be generalized to real-world data [152]. In addition, given the unrestrained availability of synthetic data (only limited by the computational power of data generation), AI models trained on synthetic data can be robust with regard to data variations [153]. Synthetic data of various types, such as tabular, text, and image, have been generated in massive quantities to train ML and DL models cost-effectively. Obesity-related data or, more generally, health-related data can be expensive to collect (eg, MRI scans) and contain confidential information (eg, patients’ names or residential addresses), which could be addressed by synthetic data generation [154].

### Human-in-the-Loop

There have been increasing concerns over AI-related data bias and ethical issues [155,156]. Fundamentally, AI models should facilitate but not replace human judgment and decision-making [157,158]. Human-in-the-loop (HITL) is an AI model that requires human interaction [159,160]. HITL ensures that algorithm biases and potentially destructive model outputs can be identified in a timely manner and corrected to prevent adverse consequences. However, such interactions between humans and machines require thoughtful designs in the data-processing pipeline, model architecture, and personnel management [159]. Data- and model-driven decision-making related to obesity, such as behavioral modifications (eg, diet or physical activity interventions) or medical treatment, can be complex [161]. AI-powered wearables and other digital health platforms can detect change in an individual’s physical activity and provide actionable information to improve health outcomes [162-164]. Mobile chemical sensors could offer timely dietary information by monitoring real-time chemical variations upon food consumption, collecting dynamic data based on an individual’s metabolic profile and environmental exposure, thus supporting dietary behavior decision-making to improve precise nutrition [165]. HITL may integrate AI model outputs with expert inputs to make informed decisions that capitalize on the strengths of both and maximize patients’ chances of health restoration and improvement [166].

### Limitations of the Scoping Review and Included Studies

To our knowledge, this study is the first to systematically review AI-related methodologies adopted in the obesity literature and project trends for future technological development and applications. However, several limitations should be noted concerning this review and the included studies. As our review focused on ML and DL methods, study-specific findings (eg, the effectiveness of an intervention and estimated associations between covariates and an outcome) were not synthesized in detail. The included studies were heterogeneous in terms of hypothesis and research question, study design, population sampled, data collection method, sample size, and data quality. The analytic approach chosen was endogenous to these study-specific parameters; therefore, across-study comparisons of model performances may not be reliable. Even within the same study, conclusions about relative model performances (eg, the prediction accuracy of logistic regression vs SVM) may lack generalizability because of the interdependency between data and ML and DL algorithms. AI technologies are rapidly advancing, with innovations and breakthroughs almost daily. A review such as this one will have a short shelf life and warrant periodic updates.

### Conclusions

This study reviewed the AI-related methodologies adopted in the obesity literature, particularly ML and DL models applied to tabular, image, and text data for obesity measurement, prediction, and treatment. It aimed to provide researchers and practitioners with an overview of the AI applications to obesity research, familiarize them with popular ML and DL models, and facilitate their adoption of AI applications. The review also discussed emerging trends such as multimodal and multitask AI models, synthetic data generation, and HITL, which may witness increasing applications in obesity research.

## Appendix

Multimedia Appendix 1 Search algorithm used in PubMed.

## Abbreviations

AGI: artificial general intelligence
AI: artificial intelligence
BFP: body fat percentage
CNN: convolutional neural network
CV: computer vision
DL: deep learning
DT: decision tree
GFA: group factor analysis
HITL: human-in-the-loop
KNN: k-nearest neighbor
LASSO: least absolute shrinkage and selection operator
MARS: multivariate adaptive regression splines
ML: machine learning
MLP: multilayer perceptron
MRI: magnetic resonance imaging
NB: naïve Bayes
NLP: natural language processing
PCA: principal component analysis
PRISMA: Preferred Reporting Items for Systematic Reviews and Meta-Analyses
PRISMA-ScR: Preferred Reporting Items for Systematic Reviews and Meta-Analyses extension for Scoping Reviews
RF: random forest
RNN: recurrent neural network
SVM: support vector machine
WC: waist circumference
WHR: waist-to-hip ratio
XGBoost: extreme gradient boosting
