# Supplementary material for: Applications of Artificial Intelligence to Obesity Research: Scoping Review of Methodologies
Source: J Med Internet Res. 2022 Dec 7;24(12):e40589. doi: 10.2196/40589 (PMC9856437; doi:10.2196/40589)
Supplement: Multimedia Appendix 1 [file jmir_v24i12e40589_app1.doc]

**Multimedia Appendix 1.** Search algorithm used in PubMed

(“artificial intelligence”[mh] OR “artificial intelligence”[tiab] OR “computational intelligence”[tiab] OR “machine intelligence”[tiab] OR “computer reasoning”[tiab] OR “machine learning”[tiab] OR “deep learning”[tiab] OR “neural network”[tiab] OR “neural networks”[tiab] OR “reinforcement learning”[tiab]) AND (“obesity”[mh] OR “obesity”[tiab] OR “obese”[tiab] OR “adiposity”[tiab] OR “overweight”[tiab] OR “body mass index”[tiab] OR “BMI”[tiab] OR “waist circumference”[tiab] OR “waist to hip”[tiab] OR “waist-to-hip”[tiab] OR “body fat”[tiab])

(The following filters were used: Humans, English, MEDLINE)
